# CHANGES IN THE MEDICARE HOME HEALTH CARE MARKET: THE IMPACT OF ACA REIMBURSEMENT POLICY

**DOI:** 10.1093/geroni/igz038.2545

**Published:** 2019-11-08

**Authors:** Jamila M Torain, Joan Davitt, Charlotte L Bright, Nancy Miller, Sarah Chard, Denise Orwig

**Affiliations:** 1 University of Maryland, Baltimore School of Medicine, Baltimore, Maryland, United States; 2 University of Maryland, Baltimore, Maryland, United States; 3 University of Maryland, Baltimore, Baltimore, Maryland, United States; 4 The University of Maryland Baltimore County, Baltimore, Maryland, United States; 5 UMBC, Baltimore, Maryland, United States

## Abstract

This study evaluated the effect of recent ACA changes to Medicare Home Health Care (HHC) reimbursements on the mix of agencies and staff in the HHC market. We used Provider of Services (POS) data and logistic regression, to determine which agency characteristics were associated with the likelihood of exiting the HHC market and likelihood of decreasing staff before (n=13,878) and after (n=13,702) implementation of the ACA-mandated reimbursement cuts. Free standing agencies had 1.35 times the odds of exiting from the HHC market post ACA cuts. There were no differences in the odds of exiting the HHC market between for-profit and non-profit agencies. Agencies in the New York, Atlanta, and Chicago regions had a greater likelihood of exiting the HHC market post ACA cuts. Small agencies had two times the odds of exiting (aOR= 2.09) and agencies with one or more branch had less than half the odds of exiting (aOR= 0.46) from the HHC market. The average number of all staff was similar before and after the ACA cuts; however, office staff and home health aides experienced the greatest decrease in number. Agencies that were for-profit, free-standing, small, and/or with one or more branch were more likely to decrease staff post the ACA cuts. Agencies in the New York, Atlanta, Chicago, Dallas and Kansas regions were more likely to decrease staff. Overall, the reimbursement cut effects varied by geographic region and had greater impact on more vulnerable agencies and staff that were non-skilled.

